# Could Dromedary Camels Develop Stereotypy? The First Description of Stereotypical Behaviour in Housed Male Dromedary Camels and How It Is Affected by Different Management Systems

**DOI:** 10.1371/journal.pone.0089093

**Published:** 2014-02-19

**Authors:** Barbara Padalino, Lydiane Aubé, Meriem Fatnassi, Davide Monaco, Touhami Khorchani, Mohamed Hammadi, Giovanni Michele Lacalandra

**Affiliations:** 1 Department of Veterinary Medicine, University of Bari, Valenzano (Bari), Italy; 2 Laboratoires d'éthologie animale et humaine EthoS -University of Rennes, Rennes, France; 3 Livestock and Wildlife Laboratory, Arid Lands Institute, Médenine, Tunisia; 4 Department of Emergency and Organ Transplantation (D.E.T.O.), Veterinary Clinics and Animal Production Section, University of Bari, Valenzano (Bari), Italy; Institut Pluridisciplinaire Hubert Curien, France

## Abstract

Dromedary camel husbandry has recently been evolving towards a semi-intensive system, due to the changes in use of the animal and the settlement of nomadic populations. Captivity could restrict its social activities, limiting the expression of various behavioural needs and causing the manifestation of stereotypy. The aims of this trial were, firstly, to identify and describe some stereotypical behaviours in captive male dromedary camels used for artificial insemination and, secondly, to study the effects on them of the following husbandry management systems: i) housing in single boxes for 24 hours (H24), ii) housing in single boxes for 23 hours with one hour free in the paddock (H23), and iii) housing in single boxes for 22 hours 30 min with 1 h of paddock time and 30 min exposure to a female camel herd (ExF). Every day, the camels were filmed in their single box in the morning for 30 minutes to record their behavioural activities and a focal animal sampling ethogram was filled in. In this study, male camels showed both oral and locomotor stereotypy most frequently when the bulls were reared in H24. Overall, this preliminary study is a starting point in the identification of stereotypies in male camels, reporting the positive effects of spending one hour outdoor and of social interaction with females.

## Introduction

Animal behaviour is influenced by the prevailing environment, and behavioural modifications are used to assess the impact of different kinds of management on animal welfare [1]. Animals housed in artificial habitats are confronted by a wide range of potentially provocative environmental challenges, and animals in captivity can develop stereotypical behaviours [2], *i.e.* repetitive, unvarying and apparently functionless behaviour patterns [3]. Since these behaviours have usually been associated with sub-optimal living conditions [4], they have often been used to assess animal welfare in different species (*e.g.* [5]–[8]). Thus, Mason & Latham [6] suggested that stereotypy should always be taken seriously as a warning sign of potential suffering. Stereotypy can take a wide range of different forms (e.g. locomotor or oral; [9]) and the causes of these abnormal behaviours have been the subject of much discussion [3], [5], [10]. The animal's lack of control over its environment, frustration, threat, fear, and lack of stimulation have all been mentioned as the main causes leading to the development of abnormal behaviour [3], [5]. One of the reasons why animals develop stereotypies is that endorphins are released when performing them, producing a form of pleasure that can help the animal to cope with the various captivity stressors, which in turn may positively reinforce the behaviour (in sows [11]; in macaques [12]).

Therefore, intensive management systems which do not allow the animal to express its behavioural needs could lead to the development of repetitive and functionless behaviours. Cooper and McGreevy [13] reported that, in horses, stereotypies were related to a number of management factors, such as concentrate feeding or social isolation, and that the form of stereotypy usually depended on the constraints to which the animals were exposed. Nicol [14] also suggested that oral stereotypies in horses (*e.g.* crib-biting, wood-chewing) may develop in response to a low-forage diet, because these behaviours may increase salivary flow, reducing gastric tract acidity and speeding up the transit of ingested feed. Locomotor stereotypy in horses (*e.g.* weaving) may derive from some frustrated attempt to move or escape from the stable [14]. Moreover, different studies have shown negative correlations between enclosure size and the prevalence of pacing in different species (red deer [15]; giraffe and okapi [16]).

The husbandry of male camels has been changing recently to more intensive management systems where they are kept isolated in a box or pen and used for programmed mating [17] or artificial insemination [18], [19]. One of the major problems of male camel rearing is that during the breeding season bulls can become very aggressive towards other males or humans and for this reason they are kept in a single box or tethered with ropes [20]. In an individual box, the animals are isolated and it is known that social isolation can create stress [21] and may lead to stereotypical behaviour. McGreevy *et al.* [22] have shown that the time spent in a single box was positively correlated with an increased risk of abnormal behaviour in horses. Camels are also social animals and in feral conditions usually live in herds and spend most of the day walking to pasture [23], so captivity could affect their behaviour, as already reported in other feral animals housed in artificial habitats [24]. Thus, our hypothesis was that, as in other species, confinement stressors such as restricted movement, reduced retreat space, forced proximity to humans, reduced feeding opportunities and maintenance in abnormal social groups, could also lead to the development of stereotypical behaviour in male dromedary camels. Since stereotypical behaviours have not yet been reported in dromedary camels, the aim of this study was to identify and describe them for the first time in males housed in single boxes.

While there are several studies on the effects of different housing systems on the behaviour of cattle, horses, hens, pigs and other domestic animals [2], [22], [25], few studies have been carried out to assess the effects of captivity in camels. Therefore, it was thought of interest to study what effects different forms of husbandry could have on their behaviour, in an attempt to suggest how to optimize camel breeding techniques in the future.

## Materials and Methods

### Animals and management systems

Four clinically healthy male dromedary camels (*Camelus dromedarius*), ranging in age from 5 to 8 years, with a mean body weight of 526±25 kg and good body condition score (3.5±0.25 arbitrary units; from 0 to 5 accordingly with Faye et al. [26]), were used for this study. All animals were identified by ear tags (#808, #514, #515, #504). The camels had been reared at the Arid Lands Institute's experimental station in Médenine, Tunisia (33°30′N, 10°40′E), 18 m above sea level.

In summer, the bulls are kept in a single open-air paddock shaded by trees whereas, starting from October, they were put into single boxes (Height = 3 m, Length = 5 m and Width = 3 m) with sand floors. They were tethered with a rope on the fetlock of the foreleg and were able to walk around inside the box. The boxes were located far from the females' pen, preventing them from seeing and touching any dams; the gates of the stable pointed eastwards, facing an open-air paddock and with a small window on the opposite wall. The gates of the stable were made by bars; camels were able to put their head outside the box through the bars or the window.

The male dromedary camels were tested in three different management systems: i) housed in single boxes for the whole day (H24; their usual and traditional method), ii) housed in the same box for 23 hours, adding 1 hour free in a paddock from 2 p.m. to 3 p.m. (H23) or iii) housed in the same box for 22 hours and 30 min with 1 hour of freedom again in the paddock from 2 p.m. to 3 p.m. and 30 minutes from 8.00 a.m. to 8.30 a.m. in boxes placed in a little pen adjacent to the female herd's pen (ExF). The paddock lies in front of the stable where the boxes are located and measures 250 square metres. Female herd's pen is bordered by a 130 cm-high wall dividing the two pens, but females were free to move and reach the males.

Each experimental condition lasted 7 days and was preceded by a habituation week, so the whole trial took six weeks (three weeks for the habituation period and three weeks of experimental situations) from February to March 2013, from the middle to the end of the breeding season, starting with the traditional husbandry form (H24) and ending with the exposure to the female rearing system (ExF).

The camels were fed with 5 kg oat hay at 9.00 a.m., and 3 kg concentrate supplement based on barley (60%), wheat bran (17.5%), olive cake (17.5%) and a mineral and vitamin complex (5%) at 3.00 p.m. The chemical composition of the oat hay was: Dry Matter (DM) = 90%, Crude Protein (CP) = 6.81%, Ash = 7.9%. Dry matter content of the concentrate was 90.9% and its chemical composition was CP = 11.4%; Acid-detergent fibre (ADF) = 13.2%; Neutral-detergent fibre (NDF) = 31.6% and Ash = 8.1%. The feeding quantity and quality remained constant during the experiment. The diet met the maintenance requirements as set by Laudadio et al. [27], and water was available once every two days.

During the trial, the bulls were used for semen collection twice weekly. They were well accustomed to this practice and to the traditional husbandry system, so we changed only the management system in accordance with the experimental protocol.

### Ethics Statement

The experiments were conducted according to the protocols approved by the Italian Ministry for Scientific Research in accordance with EC regulations. No special permission for behavioural research on wild animals such as this study is required in Italy.

### Behavioural parameters

In each management system, the four males were filmed in their single box by a video-camera (Sony Camcorder digital video) from 8.00 to 8.30 a.m. every morning for 7 days in each experimental condition, without being disturbed by the operator. The videos were analysed by an expert ethologist, who filled out a focal animal sampling ethogram, defined as the sampling method whereby the recorder chooses one individual and records all behaviours performed by the individual in a specified time window (one bull in his single box, located far or adjacent to the females' paddock for 30 minutes) [28].

The duration of the subsequent behavioural states was noted down: rumination, resting, standing, walking, looking outside and stereotypy. On the basis of the ethogram, the average time spent on these behavioural activities during the 30-min observation periods was calculated for each management system.

The videos were then studied again and after accurate analysis the presence of the stereotypical behaviours was identified and on the basis of their nature, were split into two categories:

#### Locomotor stereotypes

Head-shaking: the camel raised his head to the vertical with a very fast movement (this behaviour included a movement of the head by up to 90°). This stereotypy was considered as punctual behaviour because it lasted only about one second.

Pacing in a circle: the camel walked to the other side of his box (stopped and tried to look through a small window in the wall), and walked back to his initial position (in doing so, the camel always followed the same path which described a circle). The camel repeated this movement several times without any clear motivation: this stereotypy was considered as a state, because it always lasted more than 10 seconds.

#### Oral stereotypy

Self-biting or self-mutilation: the camel bit different parts of his own forelegs (right or left) from the shoulders to the feet. This stereotypical behaviour was considered as a state - indeed the camel could bite his legs for a variable length of time, ranging from just a few seconds to several minutes.

Bar-mouthing: licking, biting or playing with the lips on the bars of box's gate. This stereotypy was considered as punctual behaviour because it lasted only a few seconds.

Thus, a behavioural sampling ethogram, in which the observer notes all the durations and frequencies of a specific behaviour [28]) was filled out. The duration of the following behavioural states were calculated: locomotor and oral stereotypical behaviour; the total duration of stereotypical behaviours was calculated as the sum of the duration of the two categories (locomotor+oral). The frequency of the following behavioural events (punctual behaviours) was also recorded: locomotor and oral stereotypical behaviour; the total frequency of stereotypical events was also calculated as the sum of locomotor+oral. Moreover, the frequencies of putting the head outside the box and of scratching were recorded, so as to measure how many times they were stimulated by the situation outside their box and how many times they scratched, which could be a sign of boredom in captivity.

### Statistical Analyses

All behavioural parameters were subjected to repeated-measures analysis of variance using the Generalized Linear Model procedure (SAS, version 9, 1999). Independent variables were the management system (H24, H23 and ExF), the periodof observation (from Monday to Sunday), and the interaction between those variables. Data were normally distributed. Tukey's post hoc test was used to perform statistical multiple comparisons. The *p*-level was set at 0.05. All data were expressed as quadratic mean and mean standard error.

## Results

**Figure 1:**
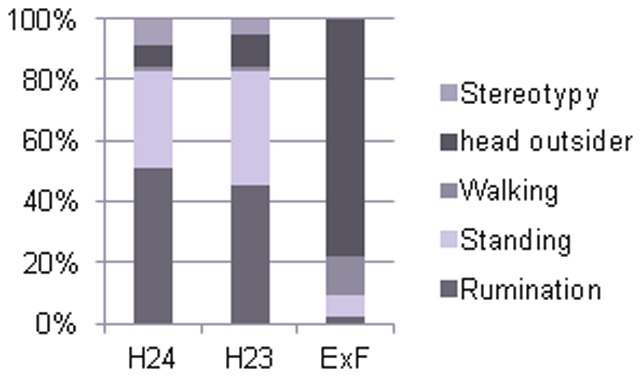
Average percentages of time spent in behavioural activities during 30: housed in single box for 24 hours (H24), housed in single box for 23 hours and one hour in paddock (H23), housed in single box for 22 hours and 30 minutes, one hour in paddock and 30 minutes of female exposition (ExF).

The average time spent in behavioural activities during 30-min observation periods while in the single box in the three different management systems (H24, H23, ExF) is reported in Fig. 1.

Three of the four male camels showed stereotypical behaviours, each differing from the others', while one of the males showed two types of locomotor stereotypy (Table 1).

**Table 1:** Description of the stereotypy shown by each camel while in their single box.

Camel	Stereotypy
808	“Bar-mouthing”: Licking, biting or playing with the lips on the bars
514	None
515	“Self-biting”: the camel bit different parts of his own forelegs (right or left) from the shoulders to the feet.
504	“Head-shaking”: the camel raised his head to the vertical with a very fast movement (this behaviour included a movement of the head up to 90°).“Pacing in a circle”: The camel walked to the other side of his box (and sometimes stopped and looked through the window), and walked again until he was back in his initial position (in doing so, the camel always followed the same path which described a circle)

The effect of the management system was significant (df = 2; F_(2,6)_ = 3.86; P = 0.02) on the frequency and the duration of the stereotypical behaviours, whereas no significant difference was observed in period (from the first to the seventh day of the week) (df = 6; F_(2,6)_ = 0.99; P = 0.44) nor in the interaction between management system and period(df = 12; F_(2,6)_ = 0.80; P = 0.64). Consequently, only the effect of the three different management systems on the behavioural parameters was considered.

**Figure 2:**
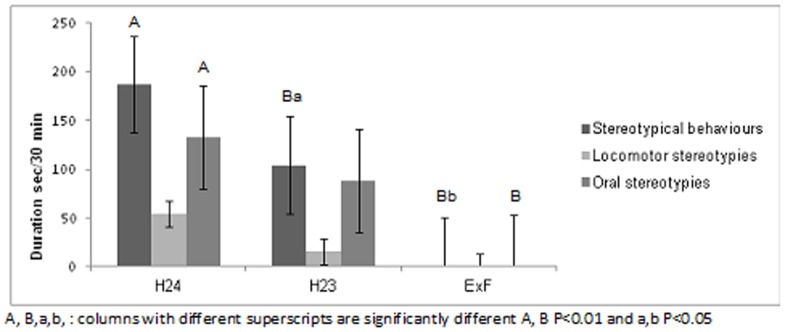
Effect of three different management systems (housed in single box for 24 hours (H24), housed in single box for 23 hours and one hour in paddock (H23), housed in single box for 22 hours and 30 minutes, one hour in paddock and 30 minutes of female exposition (ExF) on the duration (s/30 min) of stereotypical behaviour shown by male dromedary camels while in their single box. Oral stereotypies: self-biting or self-mutilation and bar-mouthing; Locomotor stereotypies: pacing in a circle; Stereotypical behaviour: sum of oral and locomotor stereotypies.

**Figure 3:**
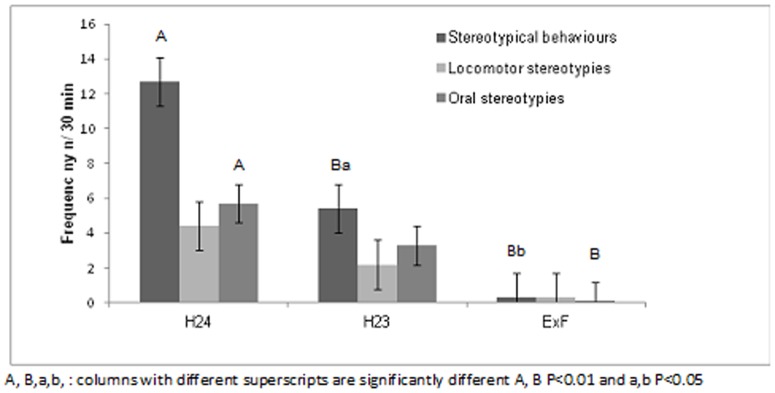
Effect of three different management systems (housed in single box for 24 hours (H24), housed in single box for 23 hours and one hour in paddock (H23), housed in single box for 22 hours and 30 minutes, one hour in paddock and 30 minutes of female exposition (ExF) on the frequency (n/30 min) of stereotypical behaviour shown by male dromedary camels while in their single box. Oral stereotypies: self-biting or self-mutilation and bar-mouthing; Locomotor stereotypies: head-shaking and pacing in a circle; Stereotypical behaviour: sum of oral and locomotor stereotypies.

The duration (in sec) of stereotypical behaviours recorded during the thirty-minute observation period every morning was highest in H24 and decreased progressively from H24 to ExF. Figure 2 shows that total stereotypical duration decreased during the weeks with one hour free in the paddock, and was significantly lower when camels were in the box adjacent to the female herd (H24 vs. ExF: 186.8±49.9 vs. 0.1±4.9 s/30 min; P = 0.03). The number of times these behavioural patterns occurred followed the same trend. When the camels were housed in H24, there was a stereotypical behaviour frequency of 12.7±1.4 in 30 min, significantly higher than for camels in systems H23 (P = 0.002) and ExF (P<0.0001); in addition, the value for H23 was also significantly higher than for ExF (P = 0.038) (Fig. 3).

**Figure 4:**
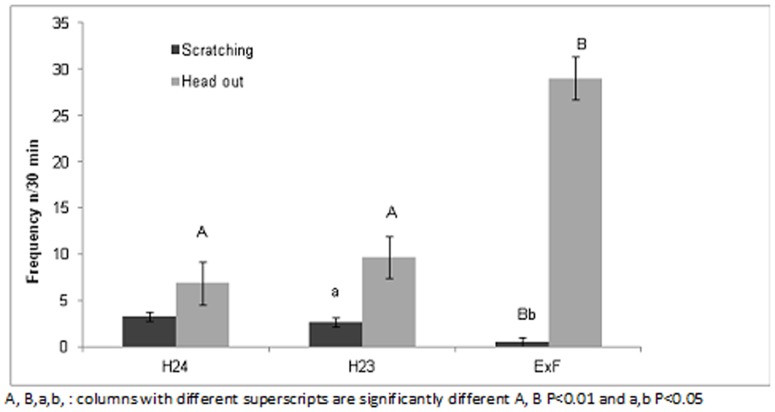
Effect of three different management systems (housed in single box for 24 hours (H24), housed in single box for 23 hours and one hour in paddock (H23), housed in single box for 22 hours and 30 minutes, one hour in paddock and 30 minutes of female exposition (ExF) on the frequency (n/30 min) of scratching and putting the head outside the box shown by male dromedary camels while in their single box.

The frequency of camels putting their heads outside their box was higher in the third housing system, when the camels were stimulated by the female herd, compared with the other two management systems (P<0.001). By contrast, the frequency of scratching behaviour was very low when they were in the pen adjacent to the female herd 0.6±0.5, and was significantly lower than for those allowed to roam free in the paddock for one hour (H23 3.0±0.9; P = 0.02) or kept in a box (H24 3.3±0.5; P = 0.003) (Fig. 4).

## Discussion

Three out of four male camels showed abnormal repeated behaviours corresponding to the general definition of stereotypical behaviours (*i.e.* repetitive, unvarying and apparently functionless behaviour patterns, [3]). As different kinds of stereotypical behaviour were observed in the three camels, a distinction was made between oral and locomotor stereotypies were distinguished as in other species [16], [9], [29], [30]. Mason et al. [31] assessed that animals could develop different repertoires of stereotypical behaviour and broke down stereotypy by taxon, to show that different orders of mammals typically favour different types of abnormal repetitive behaviour (locomotor, oral or non-locomotor body movements). This analysis revealed that stereotypical carnivora systematically prefer locomotor movements, while ungulates display oral forms. Accordingly with the data reported for ungulates, these camels developed both oral and locomotor stereotypies, showing a preference for oral ones. Indeed, two of the four camels performed different oral stereotypies: self-biting and bar-mouthing; while one of the four males performed two different locomotor stereotypies: head-shaking and pacing in a circle. The camel which exhibited head-shaking also exhibited circling, so this individual developed two different kinds of locomotor stereotypy. This finding agrees with previous observations in horses [32]: a horse already showing one locomotor stereotypy is more likely to develop a second than horses either performing oral stereotypy or expressing no form of stereotypy.

The camels in this experiment spent about 10% of the observation period stereotyping in H24; the range reported for cows is from 1% to 38% of a 24-h period before, during and after grazing [33], but in another study, where animals were reared in better conditions, this figure dropped to 1–2% [34]. A horse housed in a single box can spend up to 8 h crib-biting each day [35], whereas one female captive giraffe could spend more than 40% of the night licking and tongue-playing [36]. The latter stereotypical behaviours were related to poor management, i.e. diets with low fibre, thus confirming the effect of husbandry on the prevalence of stereotypy.

It could therefore be supposed that the traditional housing system (H24) in which camels showed the greatest incidence of stereotypies was a sub-optimal management system for this species, in agreement with studies carried out by Mills in horses [4], and that the presence of stereotypical behaviour in these individuals was a sign of poor welfare, as inferred by Mason & Latham [6] who suggested that stereotypy could be a sign of suffering. Thus, in H24 the camels were probably frustrated, lacking stimulation and control of their environment and could not therefore exhibit natural behaviours (*e.g.* it would be impossible for them to perform any social interaction), because this housing system did not satisfy the behavioural needs of this species.

These four stereotypies have already been reported in other species and different explanations have been suggested concerning the cause of these abnormal behaviours [13], [14], [16], [22]. The three major constraints were limited space, lack of stimulation (especially social contact) and controlled feeding. The development of stereotypy in these camels could also be explained by one of these three constraints or by their cumulative effect.

One of the four camels exhibited an unusual kind of oral stereotypy, *i.e.* bar-mouthing, which consisted in biting, licking or playing with the lips on the bars of the cage. The development of this stereotypy in a camel housed in a box was not surprising, indeed, these kinds of abnormal oral behaviours have also been reported in other captive animals (bank voles, [37]; pigs, [38]). Rebdo [33] suggested that feeding frustration could facilitate oral stereotypy; in our study, the camels were fed with 5 kg of oat hay and 3 kg of concentrate and did not have the opportunity to forage on pasture. Therefore, these camels may have felt feeding frustration, which could explain why one individual had developed this kind of stereotypy. This hypothesis agrees with previous studies in gilts where the time spent performing oral stereotypies (*e.g.* bar-chewing) was negatively correlated with their feed allowance (review by Lawrence & Terlouw, [38]). Nicol [14] also suggested that low-forage diets could be the main cause of the onset of oral stereotypy in horses. Moreover, Rebdo [33] has shown that heifers exhibited no abnormal behaviours when at pasture. Camels are also herbivorous animals and in natural conditions usually graze for 8–12 hours per day [23]; therefore, as has been proposed for other species, the lack of pasture may have been the trigger for this oral stereotypy.

Self-biting or self-mutilation was performed by one of the four camels during our observations. In captive-reared rhesus monkeys, the absence of physical contact with conspecifics negatively affected their behaviour and the prevalence of self-biting was positively correlated to the number of years spent in a single stall [39]. Camels are social animals and, while old males can occasionally be solitary, camels usually live in herds made up of males, females and young, or females and young without a male, or males and females without young or only one male, with an average of 25 individuals per herd [23]. Therefore, it could be supposed that social deprivation in this species may lead to the development of self-biting. McDonnell [40] suggested that social and/or feeding distraction could reduce the prevalence of self-mutilation in horses.

Pacing has been reported in a wide range of captive animals (cats, dogs, hens and horses, review by Dallaire, [30]; okapi and giraffe, [16]; red deer stags, [15]; arctic fox, polar bear, American mink, and lion, [41]; bears, [42]) and has been related to confinement-specific stressors [24]. In natural conditions, camels usually walk a lot during the day, grazing 8–12 hours daily and walking at an average speed of 2 km/h, but if necessary, they can walk 150 km per day in the desert [23]. In our study, the camels were housed in single boxes, so it is to be presumed that this area was unable to fulfil the camels' needs to walk as much during the day as they would do under natural conditions, which is probably why one camel developed this locomotor stereotypy. This hypothesis is in agreement with different studies showing a negative correlation between enclosure size and the prevalence of pacing in different species (red deer, [15]; giraffe and okapi, [16], monkeys, [3], carnivores, [41]). Moreover, Nicol [14] suggested that locomotor stereotypy in horses may derive from some frustrated attempt to move or escape from the stable, and Lawrence & Terlouw [38] supposed that the development of pacing could be based on escape behaviour. Therefore, as has been proposed for other species, providing camels with a bigger enclosure would help improve their welfare. However, Morgan and Tromborg [24] concluded that increasing the space available to the animal did not always have a positive effect on its welfare, particularly for prey animals, because it may well be that it is not the quantity of space available to the animal which is important but rather its quality, and what it gives the animals in the way of behavioural opportunity.

Head-shaking was observed in one individual. This or similar forms of stereotypical behaviour (head-tossing, bobbing, nodding or shaking) has been reported in a broad range of species (in horses, [43]; okapi and giraffe, [16]; bears, [42]; humans, [44]; rats, [45], cats, [30]; elephant, [46]). The causes of such stereotypical behaviours have been poorly investigated. According to Crowell-Davis [47], head-shaking can have a great variety of causes. As for the other stereotypies, we can suppose that the lack of stimulus, space and social contact may have led to the development of this stereotypy. Cooper et al. [48] found that increasing the visual horizon significantly decreased the prevalence of head-shaking in horses housed in single boxes (i.e. increasing visual contact between neighbours and towards the environment allows horses to monitor the environment and to interact with other horses).

Our study showed the impact of the different kinds of management on the duration and frequency of stereotypy, with more frequent stereotypical behaviour among camels kept in a box for 24 h than among those allowed 1 hour free. Therefore, we could suggest that allowing camels to walk for 1 hour daily would be a good way of improving their living conditions, rather than keeping them in a single box around the clock. Time spent stereotyping also tended to decrease between camels in groups H24 and H23, which could suggest that 1 hour free has a positive impact (or at least not a negative one) on camel welfare but it would seem to be insufficient. The duration of locomotor stereotypy decreased between H24 and ExF and this could be explained by the fact that these animals had 30 minutes more to spend in an area adjacent to the female herd where the dams could walk around as much as they wanted, stimulating the bulls.

Overall, the frequency and duration of stereotypical behaviours were higher in H24 and H23 than ExF. This suggests that exposure to females in the pen could be a better environment for male camels because it more closely matches the needs of this species (i.e. more time to walk around and more chance for social contact).

The frequency of oral stereotypy (bar-mouthing and self-biting) decreased from H24 and H23 to ExF. This is not surprising and is in accordance with our hypothesis as well as with that of McDonnell [40]: self-injuries decreased among stallions when they were placed in pasture with mares because it provided plenty of distraction and allowed the animals to perform social behaviour. In our study, camels were not placed directly with females but they could interact with and touch them (with an average of 35.9 touching events in 30 minutes), if females came near the wall and put their neck and head in the male's area, allowing contacts. Consequently, in ExF, the males put their heads outside the box (through the window or between the bars of the gate) more often than in the other groups, showing that they were monitoring their environment more when the females were close by. This could be explained by the fact that during ExF, they had a larger area of view (a wider horizon) of a more interesting environment around them than in their box, where they could only look at an empty space (poor of stimuli). Scratching was also influenced by the management system; indeed, its frequency was higher in H24 and H23 compared with ExF. According to Maestripieri et al. [49] scratching could be a sign of stress, frustration or anxiety. Moreover, Basset et al. [50] measured the frequency of self-scratching as an indicator of stress in the common marmoset (*Callithrix jacchus*) and considered an increase in this behaviour as a sign of reduced welfare. Thus, in this study, the lower frequency of scratching in ExF compared to boxed conditions could be interpreted as an improvement in their welfare needs, i.e. exposure to females could be a good way of providing male camels with stimulation and the opportunity to perform social and sexual behaviours instead of stereotypical ones.

On the basis of our preliminary findings, the traditional husbandry system of male dromedary camels reared under intensive management systems should be changed, by integrating it with at least one hour thirty minutes daily of walking around in paddocks, spending more time feeding (decreasing the concentrate/forage ratio), opening a window between the boxes which would allow the camels to have visual contact with their neighbours and spend some time near females.

## Conclusion

Male dromedary camels may develop abnormal behaviour, just as other animals do, if they live in sub-optimal conditions, and this trial was the first step in identifying locomotor and oral stereotypies in male dromedary camels housed in single boxes. Overall, this preliminary study suggests that the traditional husbandry method could be improved by allowing free movement and social contact, both of which had positive impacts on the incidence of stereotypy. Further studies are needed to identify the behavioural needs of camels reared under intensive management systems and to optimize dromedary camel welfare and breeding techniques.
